# Comparative Toxicogenomics Database (CTD): update 2023

**DOI:** 10.1093/nar/gkac833

**Published:** 2022-09-28

**Authors:** Allan Peter Davis, Thomas C Wiegers, Robin J Johnson, Daniela Sciaky, Jolene Wiegers, Carolyn J Mattingly

**Affiliations:** Department of Biological Sciences, North Carolina State University, Raleigh, NC 27695, USA; Department of Biological Sciences, North Carolina State University, Raleigh, NC 27695, USA; Department of Biological Sciences, North Carolina State University, Raleigh, NC 27695, USA; Department of Biological Sciences, North Carolina State University, Raleigh, NC 27695, USA; Department of Biological Sciences, North Carolina State University, Raleigh, NC 27695, USA; Department of Biological Sciences, North Carolina State University, Raleigh, NC 27695, USA; Center for Human Health and the Environment, North Carolina State University, Raleigh, NC 27695, USA

## Abstract

The Comparative Toxicogenomics Database (CTD; http://ctdbase.org/) harmonizes cross-species heterogeneous data for chemical exposures and their biological repercussions by manually curating and interrelating chemical, gene, phenotype, anatomy, disease, taxa, and exposure content from the published literature. This curated information is integrated to generate inferences, providing potential molecular mediators to develop testable hypotheses and fill in knowledge gaps for environmental health. This dual nature, acting as both a knowledgebase and a discoverybase, makes CTD a unique resource for the scientific community. Here, we report a 20% increase in overall CTD content for 17 100 chemicals, 54 300 genes, 6100 phenotypes, 7270 diseases and 202 000 exposure statements. We also present *CTD Tetramers*, a novel tool that computationally generates four-unit information blocks connecting a chemical, gene, phenotype, and disease to construct potential molecular mechanistic pathways. Finally, we integrate terms for human biological media used in the CTD Exposure module to corresponding CTD Anatomy pages, allowing users to survey the chemical profiles for any tissue-of-interest and see how these environmental biomarkers are related to phenotypes for any anatomical site. These, and other webpage visual enhancements, continue to promote CTD as a practical, user-friendly, and innovative resource for finding information and generating testable hypotheses about environmental health.

## INTRODUCTION

The Comparative Toxicogenomics Database (CTD; http://ctdbase.org/) provides curated content relating chemical exposures with genetic, molecular, and biological outcomes to help understand and formulate testable hypotheses regarding environmental health (1). Chemical, gene, phenotype, anatomy, disease, taxa and exposure data are manually curated from the scientific literature by CTD biocurators using controlled vocabularies and ontologies (2), enabling data comparison across species and providing transparency and traceability for the user. CTD implements several efficient protocols to facilitate manual curation, including the use of a web-based curation application tool for data entry with automatic quality control mechanisms (2), text mining and article ranking to prioritize workflows (3), targeted journal curation for increased data currency (4) and chemical-centric curation for data completeness (4). CTD provides a comprehensive suite of search functionality (http://ctdbase.org/search/), analytical tools (http://ctdbase.org/tools/), and download files (http://ctdbase.org/downloads/). To maximize interoperability with other resources, CTD is committed to data FAIRness (5) by transparently implementing and documenting our data policies (http://ctdbase.org/about/ctdDataFairness.jsp), maintaining compliance with reporting standards set by the FAIRsharing information resource (6), and registering with both BioDBcore (https://fairsharing.org/biodbcore-000173/) and the Nucleic Acids Research Molecular Biology Database Collection (http://www.oxfordjournals.org/our_journals/nar/database/summary/1188).

CTD functions as both a knowledgebase (reporting content directly curated from the scientific literature) and a discoverybase (generating predictions made possible by the integration of diverse data). The success of this dual nature is dependent not only upon CTD being continually updated with the latest relevant information, but also on our ability to provide unique functionalities that help scientists discover novel connections. Here, in this biennial update, we highlight CTD’s increased data content and a major new tool that generates CGPD-tetramers (four-unit information blocks linking a Chemical, Gene, Phenotype and Disease) that can be leveraged to address knowledge gaps and construct potential chemical-disease mechanistic pathways.

## NEW FEATURES

### New content: updated CTD statistics

CTD is updated every month with content from newly curated articles (http://ctdbase.org/about/dataStatus.go). The selection of articles for CTD manual curation is chemical-centric: we target the literature based upon the mention of a chemical in a paper, employing methods to ensure both increased data currency and data completeness for chemicals (4). Consequently, the curation of all associated gene, phenotype, anatomy, and disease content is secondary to and dependent upon the chemical mentioned in any given article. As of August 2022, CTD includes over 3.4 million evidence-based manually curated chemical–gene, chemical–phenotype, chemical–disease, gene–disease and chemical–exposure interactions, reflecting a 20% increase in curated content since our last update (7). These interactions relate information for 17 117 chemicals, 54 355 genes, 6187 phenotypes, 954 anatomical terms and 7274 diseases from 622 comparative organisms. Internal integration of these direct interactions generates >31 million gene-disease and 2.9 million chemical-disease predictive inferences that are statistically ranked (8). External integration of CTD content with imported annotations from the Gene Ontology (GO) (9), KEGG (10), Reactome (11) and BioGRID (12) produces an additional 13 million inferences. In total, CTD includes over 50 million toxicogenomic relationships for computational analysis and hypothesis development.

CTD Exposure is a sophisticated annex module that captures detailed information from articles describing real-world exposure stressors, events, measurements, and outcomes to help characterize the exposome (13,14). This complex exposure content is seamlessly integrated with CTD’s core curation of chemical, gene, phenotype, and disease interactions, addressing the scientific community's urgent need to couple the exposome concept to mechanistic toxicology (15–17). For CTD Exposure, curators survey and capture a large array of data types, including chemical stressors, environmental sources, receptor demographics (including age, gender, smoking status, health, and genotypes), levels of environmental biomarkers found in biological media, geographic locations, exposure timeframes, and adverse outcomes, to name a few. As of August 2022, the CTD Exposure module reports 202 243 manually curated exposure statements from 3259 exposure studies, representing a 24% increase in statements since our last report (7), and includes data for 1492 environmental chemical stressors, 868 human genes, and 966 exposure outcomes (478 phenotypes and 488 diseases).

To gauge the value of CTD to the community, we internally track two metrics: citation indices and database linkage. Cumulatively, CTD has over 3930 total citations, and since 2021 is cited at a rate of two citations per day (i.e. every 12 h a new paper is published that mentions the use of CTD in the study). Additionally, 192 external databases now link to and/or reuse CTD information at their own resource (http://ctdbase.org/about/publications/#use), a 32% increase since our last update; this helps to further disseminate CTD content to diverse scientific communities.

### New tool: CTD tetramers

**Figure 1.**
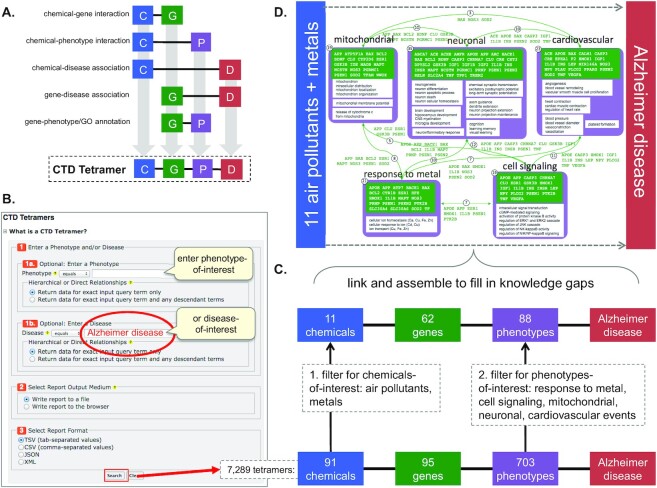
New *CTD Tetramer* tool generates CGPD-tetramers that can help fill in knowledge gaps and construct potential molecular mechanistic pathways. (**A**) A CGPD-tetramer is a computationally generated information block composed of four units: an initiating chemical (C), an interacting gene (G), a modulated phenotype (P), and a disease (D) outcome. To generate a tetramer, five direct dyad evidence statements are integrated from CTD: C–G interaction, C–P interaction, C–D association, G–D association, and an imported G–P annotation, since GO biological process terms are the equivalent vocabulary for phenotypes in CTD (19). A tetramer will be generated only if all five direct dyad evidence statements currently exist in CTD. This computational process generates a selective, but supported, set of tetramers and, importantly, does not require *a priori* knowledge by the user. (**B**) The *CTD Tetramer* tool (http://ctdbase.org/tools/tetramerQuery.go) can be queried for any phenotype and/or environmental disease-of-interest to automatically generate all possible tetramers. (**C**) For Alzheimer disease, the tool generates 7289 tetramers, composed of 91 chemicals, 95 genes, and 703 phenotypes. This output can be manually sorted, surveyed and filtered to focus on any subset of chemicals-of-interest (here, air pollutants and metals) as well as phenotype clusters (e.g. response to metal, cell signaling, mitochondrial-related, neuron-related, and cardiovascular-related), resulting in a sub-set of 601 tetramers, composed of 11 chemicals, 62 genes and 88 unique phenotypes. (**D**) Users can manually assemble the tetramers by hand by linking them together using the shared genes (green boxes/text/arrows) that connect different phenotype clusters (purple boxes) to build a complex, interrelated map. This manual process, outlined in (21), fills knowledge gaps with potential molecular mechanistic steps (e.g. intermediate genes and phenotypes) that link air pollution/metal exposure to Alzheimer disease, producing a testable framework for experimental verification.

As indicated above, CTD integrates a variety of data-types to transform the curated knowledgebase into a discoverybase. This approach computationally identifies potential intermediate actors that can link different data-types. For example, Gene Inference Networks identify genes that can fill the gaps between a curated chemical–disease or chemical–phenotype association (8), providing a potential molecular mechanism connecting exposure to adverse outcomes; similarly, Chemical Inference Networks describe chemicals that can help inform gene–disease or phenotype–disease relationships (8). As a discoverybase, CTD has been successfully leveraged to construct mode-of-action or adverse outcome pathways for bisphenol A and lung cancer (18), arsenic and male reproductive toxicity (19), and 4-nonylphenol and Parkinson disease (20), amongst many others. Recently, CTD described another novel integration strategy (21) that combines five curated dyad interactions (chemical–gene, chemical–phenotype, chemical–disease, gene–disease and gene–phenotype) to generate CGPD-tetramers: a reductionist unit of computational information that links together an initiating chemical, an interacting gene, a modulated phenotype, and a disease outcome (Figure 1A). At CTD, we operationally distinguish ‘phenotype’ from ‘disease’, with phenotype referring to a non-disease biological process (e.g. cell population proliferation) vs. a disease term-based endpoint (e.g. ovarian cancer). CTD tetramers provide potential insight into molecular mechanisms and can be assembled to construct complex chemical-induced pathways that help fill the knowledge gaps for environmental heath studies, as demonstrated in case studies for air pollution-associated cardiovascular disease (21), the role of cadmium in Alzheimer disease (22), neurological health risks associated with pesticide residues in medical cannabis (23), and respiratory outcomes from exposure to Juul e-cigarettes (24).

We created the new web-based tool *CTD Tetramers* (http://ctdbase.org/tools/tetramerQuery.go) that enables users to easily generate their own CGPD-tetramers for any environmental disease- or phenotype-of-interest (Figure 1B). For example, querying for ‘Alzheimer disease’ generates 7289 tetramers composed of 91 chemicals, 95 genes, and 703 phenotypes. The results can be manually surveyed for specific chemicals-of-interest (e.g. 11 air pollutants and metals) and phenotype categories-of-interest (e.g. response to metals, cell signaling, mitochondrial events, neuronal events, and cardiovascular events) to filter the data set to 601 tetramers composed of 11 chemicals, 62 genes and 88 phenotypes (Figure 1C). Finally, researchers can manually consolidate the tetramers to generate extended chemical-disease pathways by hand. We previously described our manual method (21) wherein similar phenotypes are first binned into groups and then tetramers are aligned based upon connecting genes shared between the phenotype bins. Such manually constructed maps can fill knowledge gaps with specific, potential molecular intermediates. Here, chemical exposure to air pollutants and metals can be connected to Alzheimer disease through a set of linked genes and phenotypic biological processes, progressing from molecular to cellular to system levels (Figure 1D). These predictive, hand-drawn maps can serve as frameworks for verification and refinement and can help inform the design of adverse outcome pathways (25–27).

### New connections: CTD exposure and CTD anatomy

**Figure 2.**
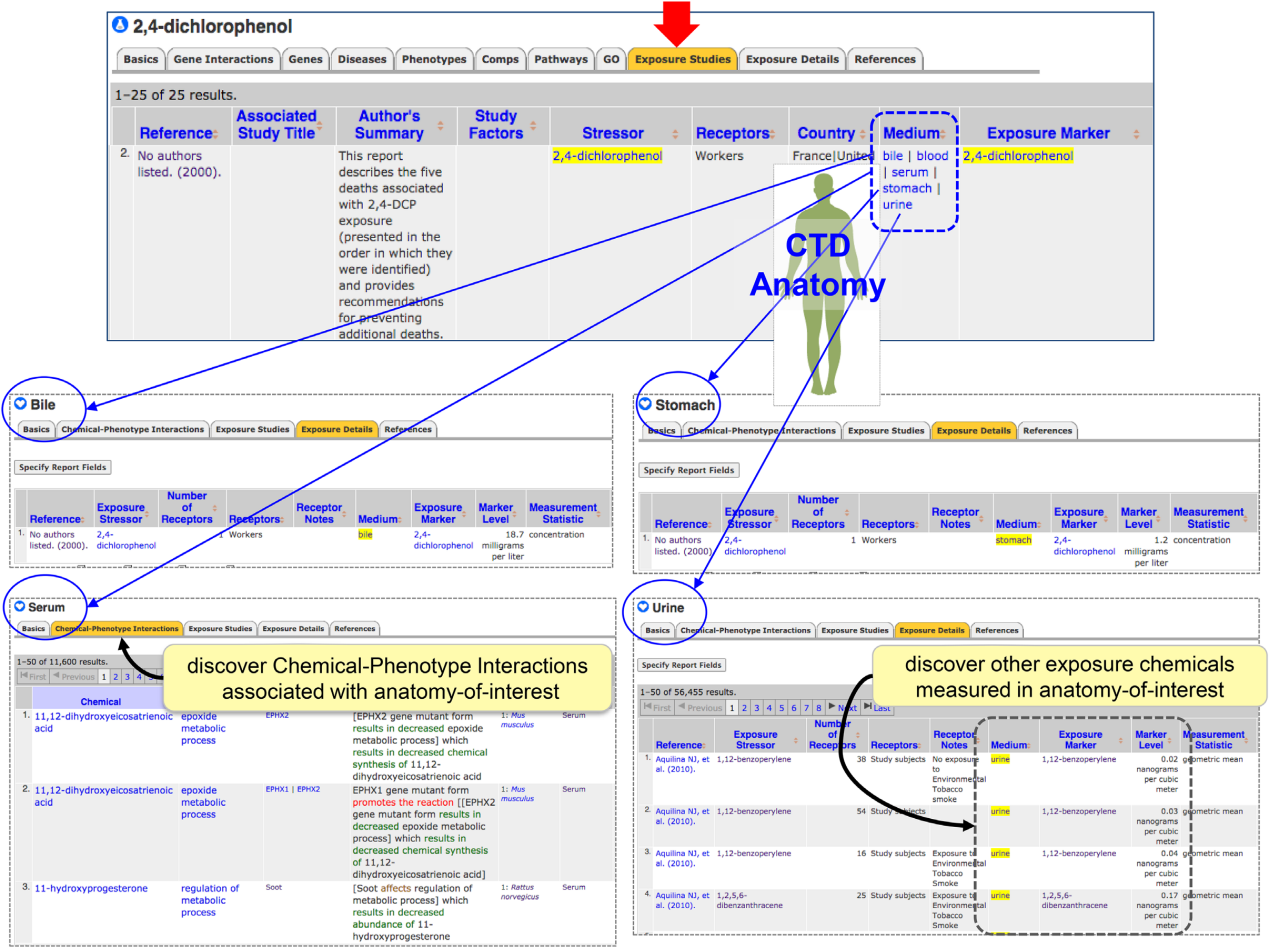
Human biological media assayed for chemical biomarkers in CTD Exposure are now integrated with CTD Anatomy. An exposure study reports that the environmental chemical 2,4-dichlorophenol is measured in a variety of human media (here, bile, blood, serum, stomach, and urine). These terms are now linked to their corresponding pages in CTD Anatomy, allowing users to seamlessly traverse and find additional chemicals detected in the same media reported by other exposure studies, as well as peruse the chemical-induced phenotypes associated with them. This integration helps tie mechanistic toxicology to the exposome concept.

Previously, we described CTD Anatomy pages (http://ctdbase.org/voc.go?type=anatomy) that organize chemical-induced phenotypes from an anatomical perspective (28). As part of the CTD Exposure module (13,14), curators collect real-world measurements of chemical exposure biomarkers in commonly surveyed human biological media (e.g. blood, plasma, serum, urine, hair, nails, saliva, adipose, semen, skin, patella, lung, etc). These human media are now mapped and linked to their corresponding CTD Anatomy terms, expanding the capacity to search and analyze exposure data within an anatomical context. For example, an environmental phenol is reported in urine, bile, serum, and stomach (Figure 2), and now users can easily identify other chemicals measured in those same human media, as well as navigate to those chemicals or peruse the chemical-induced phenotypes associated with them in any specific human medium. Currently, >75 diverse human media surveyed in exposure studies (including pregnancy-related terms such as amniotic fluid, cord blood, breast milk, colostrum, placenta, meconium and umbilical cord) are integrated with CTD Anatomy pages, providing potential mechanistic insight for exposome models.

### New visualization: streamlined webpages

With the rapidly expanding content in CTD, it has become necessary to make webpage viewing and scrolling more organized and productive. To aid in such visualization, we condensed *Inference Network* and other aggregate columns on CTD webpages to display only the number of aggregated data-types and include an expand/collapse button (+/–) that users can toggle to see the data used to make the inference or other association. For example, the CTD webpage listing phenotypes inferred to myocardial infarction (https://bit.ly/CTDMIphenotypes) is now presented in a more condensed version. With both the *Chemical Inference Network* and *Gene Inference Network* columns collapsed, the overall content can be more readily surveyed, assessed, and scrolled through. The inference networks are viewable by simply clicking the expand/collapse buttons found next to the summary text. Similar collapsed *Inference Networks* are also found for the relevant data-tabs on all Gene, Chemical, Phenotype, and Disease webpages in CTD, streamlining the overall look and feel to these pages.

## FUTURE DIRECTIONS

CTD’s foremost objective is to facilitate understanding of the complex connections between environmental exposures and human health. To this end, we will continue curating the scientific literature to increase data content, improve data completeness, and maintain data currency. This ensures CTD is relevant, comprehensive, and up-to-date: critical requirements for a knowledgebase and informative discoverybase. We also plan on exploring ways to provide additional functionality to the *CTD Tetramers* tool, such as allowing users to start the query with a chemical-of-interest (in addition to the existing phenotype- and or disease-based functionality) to generate tetramers for that compound.

## SUMMARY

1. CTD manually curated content increased by 20% and, when integrated with other data-types, now includes 50 million toxicogenomic relationships.
2. *CTD tetramers* is a new analytical tool that computationally generates CGPD-tetramers on-demand for any phenotype or environmental disease, facilitating the construction of potential molecular mechanistic and adverse outcome pathways.
3. New integration links between the CTD exposure and CTD anatomy modules allow users to seamlessly navigate to exposure chemical profiles for different anatomical terms and help tie mechanistic toxicology to the exposome concept.
4. CTD webpages are streamlined with collapsible information, enhancing overall page visualization and content assessment.

## DATA AVAILABILITY

CTD content is available from http://ctdbase.org/ and files can be downloaded from http://ctdbase.org/downloads/. To cite CTD data, please see: http://ctdbase.org/about/publications/#citing. If you are interested in establishing links to CTD data, please notify us (http://ctdbase.org/help/contact.go) and follow the instructions (http://ctdbase.org/help/linking.jsp). External resources using CTD content are collected and highlighted (http://ctdbase.org/about/publications/#use).
